# Supplementary material for: The Application of Bacillus subtilis for Adhesion Inhibition of Pseudomonas and Preservation of Fresh Fish
Source: Foods. 2021 Dec 13;10(12):3093. doi: 10.3390/foods10123093 (PMC8701017; doi:10.3390/foods10123093)
Supplement: Supplementary file 1 [file foods-10-03093-s001.zip › foods-1455470-supplementary.pdf]

foods

MDPI

Supplementary Materials

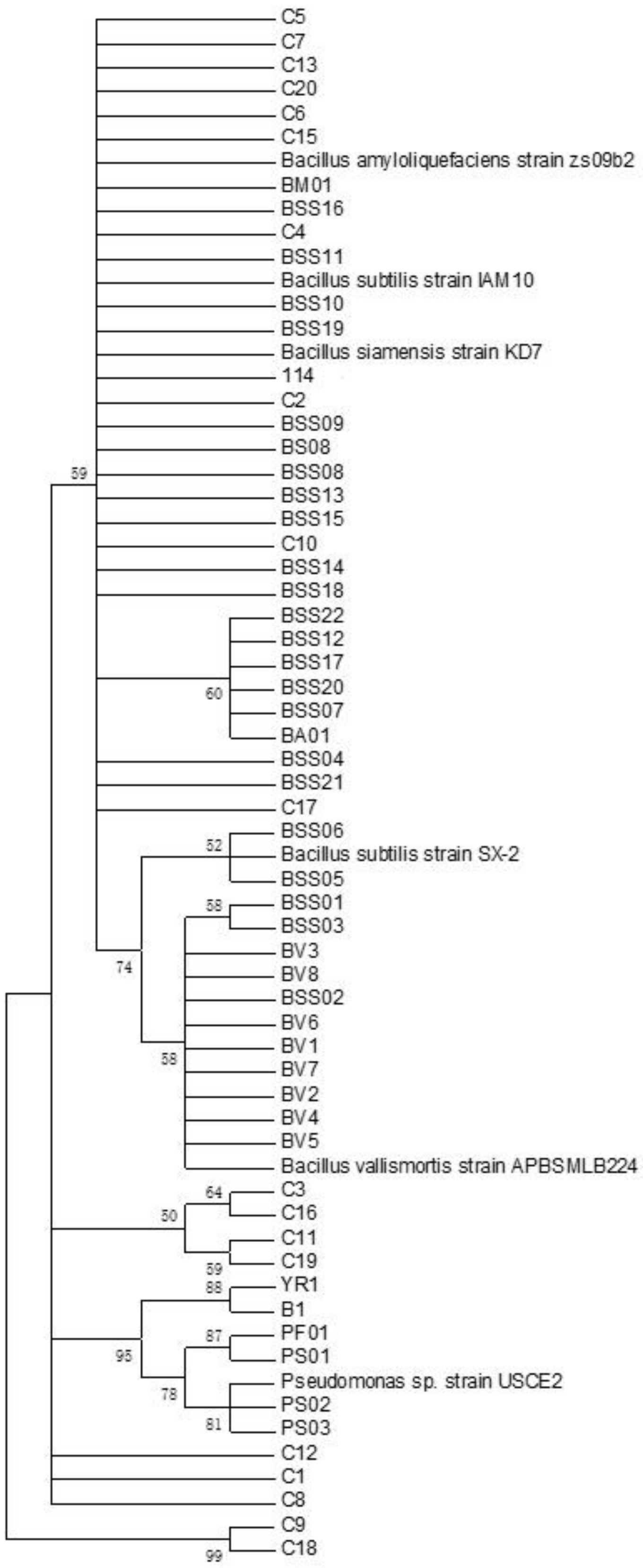

Figure S1. Phylogenetic tree analysis plot of 56 bacterial strains isolated from tilapia body and its living environment was carried out.

Table S1. Determination of volatile flavor compounds in fish flesh after storage for 0 and 8 days by GC-MS.

No.	Compounds	Concentration（mg/L）							
		**Con_0**	**H_0**	**M_0**	**L_0**	**Con_8**	**H_8**	**M_8**	**L_8**
		Alcohols							
F1	Ethanol	2.429	0	1.990	2.418	0	0	0	0
F2	Phenylethyl Alcohol	0	0	0	0	0.049	0.072	0	0
F3	1-Octen-3-ol	0	0.053	0.045	0.045	0.113	0.085	0.085	0.077
F4	1-Propanol, 3-(methylthio)-	0	0	0	0	0.039	0	0	0
F5	1-Butanol, 3-methyl-	0	0	0	0	0.268	0	0	0
F6	1-Hexanol, 2-ethyl-	0	0.103	0.103	0.053	0	0	0	0.082
F7	1-Octanol	0	0	0	0.045	0	0.025	0	0
	Total alcohols	2.429	0.156	2.138	2.561	0.469	0.182	0.085	0.159
		Alkanes							
F8	Benzene, 1,1-butylidenebis-	0	0	0	0	0	0.03	0	0
F9	Benzene, 1,1-pentylidenebis-	0.043	0	0	0	0	0	0	0
F10	Cycloheptane	0	0	0	0	0	0	0.011	0
F11	Heptadecane, 2,6-dimethyl-	0	0	0	0	0	0.026	0.047	0
F12	Nonadecane, 9-methyl-	0.032	0	0	0	0	0	0	0
F13	Dodecane	0.084	0.063	0.050	0.176	0.026	0	0	0.061
F14	Tridecane	0.029	0	0	0	0.034	0.033	0.033	0
F15	Tetradecane	0.062	0	0.026	0.123	0.031	0.02	0.034	0.051
F16	Pentadecane	0.193	0.042	0.072	0.121	0	0	0.128	0.065
F17	Pentadecane, 3-methyl-	0	0	0	0	0.023	0	0	0
F18	Pentadecane, 2,6,10,14-tetramethyl-	0.046	0	0.038	0.064	0.022	0	0	0.032
F19	Hexadecane	0.082	0.013	0.023	0.094	0.032	0.107	0.046	0.023

Continuation Table S1

No.	Compounds	Concentration（mg/L）							
		**Con_0**	**H_0**	**M_0**	**L_0**	**Con_8**	**H_8**	**M_8**	**L_8**
F20	Heptadecane	0.231	0.016	0.101	0.090	0.045	0.106	0.187	0.05
F21	Octadecane	0	0	0	0	0	0	0.036	0
F22	Oxirane, hexadecyl-	0	0	0	0	0	0	0	0.025
F23	Nonadecane	0	0	0	0	0	0.032	0.036	0
F24	Eicosane	0	0	0	0	0.037	0.17	0.061	0
	Total alkanes	0.802	0.134	0.310	0.670	0.250	0.524	0.619	0.307
	Olefins								
F25	1,3-Cyclooctadiene	0	0	0.023	0	0	0.056	0.04	0
F26	4,8,12-Tetradecatrienal, 5,9,13-trimethyl-	0	0	0	0	0	0.043	0	0
F27	1,3-trans,5-cis-Octatriene	0	0.016	0	0	0	0	0	0
F28	Z-8-Hexadecene	0	0	0	0	0.016	0	0	0
F29	5-Octadecene, (E)-	0	0	0	0	0	0.104	0	0
	Total olefins	0	0.016	0.023	0	0.016	0.203	0.040	0
	Ethers								
F30	Ethanol, 2-(2-ethoxyethoxy)-	0.026	0	0.015	0.021	0.021	0.033	0.02	0.019
	Aldehydes								
F31	Benzaldehyde	0.093	0.059	0.046	0.176	0.27	0.184	0.126	0.102
F32	3,5-di-tert-Butyl-4-hydroxybenzaldehyde	0	0	0	0	0.01	0	0	0
F33	Methional	0	0	0	0	0.022	0	0	0.019
F34	Hexanal	0	0.132	0	0	0	0	0.377	0.229
F35	Heptanal	0	0	0	0	0.071	0	0.058	0
F36	Octanal	0	0	0	0	0.078	0	0.044	0
F37	Nonanal	0.118	0.117	0.075	0.098	0.195	0.08	0.137	0.109
F38	Decanal	0	0	0	0	0.162	0.09	0	0
F39	Pentadecanal	0	0	0	0	0	0.17	0.108	0.052

Continuation Table S1

No.	Compounds	Concentration（mg/L）							
		Con_0	H_0	M_0	L_0	Con_8	H_8	M_8	L_8
F40	Hexadecanal	0	0	0.098	0	0	0	0	0
F41	Tetradecanal	0.027	0	0	0	0	0	0	0.013
F42	Hexadecanal	0	0	0	0	0.231	0.089	0.183	0
	Total aldehydes	0.238	0.308	0.220	0.274	1.039	0.613	1.033	0.524
	Acids								
F43	Benzoic acid	0	0.030	0	0	0	0	0	0
F44	Pentanoic acid, 2,2,4-trimethyl-3-carboxyisopropyl, isobutyl ester	0	0	0	0	0.029	0	0	0
F45	6-Octadecenoic acid	0	0.027	0	0	0	0	0	0
	Total acids	0	0.057	0	0	0.029	0	0	0
	Esters								
F46	Hexanedioic acid, dimethyl ester	0	0	0.008	0	0.016	0	0	0.013
F47	Carbamodithioic acid, diethyl-, methyl ester	0.085	0	0.050	0	0	0	0	0
F48	Hexadecanoic acid, methyl ester	0	0	0	0	0.088	0.073	0	0
F49	Hexadecanoic acid, ethyl ester	0.086	0	0.099	0	0.03	0	0	0
F50	Tetradecanoic acid, ethyl ester	0.039	0	0.042	0	0	0	0	0
F51	Ethyl 9-hexadecenoate	0	0	0	0	0.026	0	0	0.025
F52	Ethyl Oleate	0.035	0	0.037	0	0	0	0	0
F53	E-11-Hexadecenoic acid, ethyl ester	0.104	0	0	0	0	0	0	0
F54	Di-sec-butyl phthalate	0	0	0	0	0.036	0	0	0
F55	1,2-Benzenedicarboxylic acid, bis(2-methylpropyl) ester	0	0	0	0	0	0.024	0	0
	Total esters	0.350	0	0.237	0	0.196	0.097	0	0.038
	Phenols								
F56	Phenol	0	0	0	0	1.77	0.22	0.488	0.381
F57	Phenol, 2,4-bis(1,1-dimethylethyl)-	0.676	0.303	0.828	0	0.66	0.523	0.444	0.389
F58	Butylated Hydroxytoluene	0.045	0.037	0.026	0.033	0	0	0.073	0.045

Continuation Table S1

No.	Compounds	Concentration（mg/L）							
		**Con_0**	**H_0**	**M_0**	**L_0**	**Con_8**	**H_8**	**M_8**	**L_8**
F59	Phenol, 4-(1,1,3,3-tetramethylbutyl)-	0.105	0.038	0.046	0	0	0	0	0
	Total phenols	0.826	0.378	0.900	0.033	2.430	0.743	1.005	0.815
	Ketones								
F60	Acetophenone	0	0	0	0	0.013	0	0	0
F61	5,9-Undecadien-2-one, 6,10-dimethyl-, (E)-	0	0	0	0	0.042	0.141	0.065	0
F62	2-Nonanone	0	0	0	0	0	0	0.015	0
F63	2-Pentadecanone, 6,10,14-trimethyl-	0	0	0	0	0.027	0	0	0
F64	2,3-Octanedione	0	0.043	0	0	0	0	0.095	0.06
F65	Bicyclo[3.2.0]heptan-2-one	0	0.008	0	0	0	0	0	0
	Total ketones	0	0.051	0	0	0.082	0.141	0.175	0.060
	Heterocyclic								
F66	Benzene, (1-butyloctyl)-	0	0	0	0	0	0.062	0	0
F67	Benzene, 1,3-bis(1,1-dimethylethyl)-	1.637	1.253	1.199	2.425	1.4	0.581	0.878	1.81
F68	Naphthalene	0.113	0.089	0.083	0.106	0.065	0.087	0.076	0.054
F69	Naphthalene, 1,2,3-trimethyl-4-propenyl-, (E)-	0.061	0.049	0.049	0	0.017	0.148	0	0.066
F70	Oxime-, methoxy-phenyl-	0.140	0.335	0.103	0	0.14	0	0.089	0.302
F71	2-Acetylfluorene	0	0	0.021	0	0.034	0	0	0
F72	Piperazine, 2-methyl-	0.037	0	0	0	0	0	0	0
F73	Indole	0	0	0	0	0.057	0.017	0.027	0.039
F74	Thiophene, 2,5-diethyl-	0	0	0.021	0	0	0	0	0
	Total heterocyclic	1.987	1.726	1.476	2.531	1.713	0.895	1.070	2.271
	Amines								
F75	Formamide, N, N-dibutyl-	0	0	0	0	0.091	0	0	0
F76	Methyl-(9-oxa-bicyclo [3.3.1] non-6-en-2-yl)-amine	0	0	0	0	0	0	0.065	0
	Total amines	0	0	0	0	0.091	0	0.065	0

Continuation Table S1

No.	Compounds	Concentration（mg/L）							
		**Con_0**	**H_0**	**M_0**	**L_0**	**Con_8**	**H_8**	**M_8**	**L_8**
	Total	6.659	2.826	5.318	6.090	6.336	3.431	4.112	4.193

Table S2. Key flavor compounds and their odor characterization based on PLS-DA model.

No.	Compounds	Odor characteristics
F73	Indole	Fecal odor
F56	Phenol	Plastic, rubber
F5	1-Butanol, 3-methyl-	Spicy, fermented grain fruit flavor
F46	Hexanedioic acid, dimethyl ester	Nutty
F48	Hexadecanoic acid, methyl ester	Grease, waxy
F33	Methional	Moldy potatoes, vegetables
F37	Nonanal	The scent of lemons, flowers
F42	Hexadecanal	The faint scent of flowers and waxy
F35	Heptanal	Herbaceous, fruity, nutty
F36	Octanal	
F51	Ethyl 9-hexadecenoate	Waxy, fruity, creamy
F38	Decanal	The scent of orange peel and tangerine peel
F3	1-Octen-3-ol	The smell of fresh, bitter almonds
F31	Benzaldehyde	Sweet and bitter nutty, woody
F57	Phenol, 2,4-bis(1,1-dimethylethyl)-	Odor of phenol and alkylphenol
F40	Hexadecanal	Cardboard
F49	Hexadecanoic acid, ethyl ester	Waxy fruity creamy milky balsamic
F50	Tetradecanoic acid, ethyl ester	Sweet, waxy
F68	Naphthalene	Pungent taste
F34	Hexanal	Fresh green fatty aldehydic grass
F13	Dodecane	A mild, flat smell
F15	Tetradecane	
F16	Pentadecane	
F18	Pentadecane, 2,6,10,14-tetramethyl-	
F19	Hexadecane	
F20	Heptadecane	
F23	Nonadecane	
F25	1,3-Cyclooctadiene	—
F30	Ethanol, 2-(2-ethoxyethoxy)-	—
F1	Ethanol	Pungent odor
F67	Benzene, 1,3-bis(1,1-dimethylethyl)-	Slight malodor
F71	2-Acetylfluorene	—
F27	1,3-trans,5-cis-Octatriene	—
F45	6-Octadecenoic acid	Pungent odor
F70	Oxime-, methoxy-phenyl-	Fishy smell
